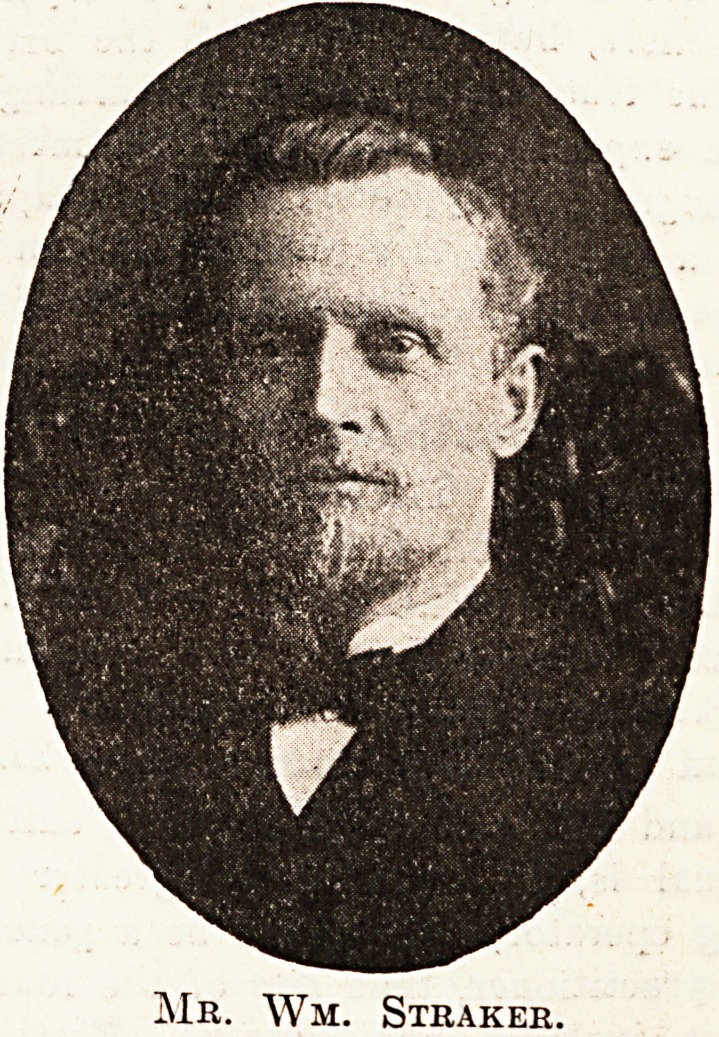# The Newcastle Conference: The Place and Personalities of the Week

**Published:** 1914-06-20

**Authors:** 


### THE NEWCASTLE CONFERENCE.

### The Place and Personalities of the Week.

Whether by accident or design, the members of the British Hospitals' Association have been singularly fortunate in the different places at which the conferences have successively been held. Birmingham, Oxford, and Newcastle, to take the last three, are not only centres of three different types of hospital work, but they also represent three varied, but characteristic, phases of the great modern city. As a *venue* for a conference of this kind Newcastle could hardly be beaten, and those who have been compelled to stay away may well envy the conditions under which it is being held. The library of the Royal Victoria Infirmary, Newcastle, an illustration of which we are enabled to publish by the courtesy of the *Architectural Review*, is no anti-climax even to the buildings at Oxford, and the meetings which will take place in it could have no more desirable accommodation. The first illustration, of Leazes Park, which adjoins the grounds of the infirmary, is where the Lord Mayor's garden party was held on Thursday afternoon, and gives some idea of the fine situation which was made use of when the institution was rebuilt in 1906. We may briefly recall that the institution, which is built on the pavilion plan from designs by Mr. Percy Adams, F.R.I.B.A., and Mr. W. Lister Newcombe, F.R.I.B.A., of Newcastle, consists of an immense corridor, from which branch off in front an administration block with two long pavilions and two short ones on either side, all at right angles to the corridor; and at the rear four long pavilions and two short ones, the latter at either end. At the right front extremity of the administration block is the library.

Sir George Hare Philipson, M.D., D.C.L., J.P.

General View from Leazes Park, where the Garden Party took place.

Mr. George Haliburton Hume, M.D., D.C.L., F.R.C.S.

Fortunately, we are also able to give illustrations of the chief personalities at the Conference, of whom mention must first be made of Councillor Johnstone Wallace, J.P., the Lord Mayor of Newcastle, who is acting as president of the Conference. By the time that this issue of The Hospital is in the hands of our readers the members of the Association will have already heard his address, which has been looked forward to with more than ordinary interest from the fact that his abilities have led him to make a study of the problems connected with hospitals and industries in the North, and therefore will have provided him with an opportunity of having stepped outside the formal limits of an official address of welcome.

The onus of the arrangements has inevitably fallen upon Mr. Roden H. P. Orde, the house governor and secretary of the Royal Victoria Infirmary, who has acted as local secretary of the conference. His successful career began at Cambridge, where he rowed in the Eight, and—shadow of future responsibilities!—acted as secretary to the Cambridge University Boat Club. Soon after leaving the 'Varsity, with, unlike many rowing men, a B.A. degree, he was introduced, we believe by his friend, Mr. G. Q. Roberts, to Sir Edmund Hay Currie. He thereupon acted as one of Sir Edmund's assistants at his Engineering College at Folkestone. The college, however, had a somewhat chequered career, though it is only fair to add that on moving to Seafield, after Folkestone had been given up, it flourished, and is still there to-day. On its move, however, Mr. Orde went to South Africa, and was engaged in work on the Rand. To this, however, the Jameson Raid put an end, and he returned to England, where a post was found in the house governor's office of the London Hospital. It was here that his hospital career officially began. Being extremely keen on his work, he mastered the details of all that he had to do, and when he was appointed secretary and house governor of the Newcastle Infirmary his chiefs in London were as much sorry for themselves as they were glad on his account. Mr. Orde's experiences here and abroad have produced a combination of determination with a conciliatory manner, which, it needs no emphasis, is as valuable as it is rare among those who have to deal with committees and the representatives of large organisations. These qualities, anyway, were sufficient to establish him firmly in his new post, which, oddly or inevitably according to how the

Mr. Roden H. P. Orde.

Sir Riley Lord, J.P.

The Library.

fact strikes you, took him back to the neighbourhood of his old home. The present Lord Armstrong, who has been one of the chief benefactors of the Newcastle Royal Infirmary, was a boy friend of Mr. Orde's, and, no doubt, the fact of his early association with the locality has enabled him to get into personal touch with various people, to the great advantage of the institution.

Except for his secretarial experience in connection with the C.U.B.C., Mr. Orde's training in hospital management and accounts was received in full at the London Hospital, where he was as much valued by the committee as by his immediate chief.

Mr. G. H. Hume, M.D., F.R.C.S., Vice-President, is well known to Newcastle as consulting physician to the Royal Victoria Infirmary, and to a wider public still as author of a comprehensive and

THE LORD MAYOR OF NEWCASTLE-ON-TYNE.

admirably written history of the institution. He has practised in Newcastle for forty-six years, and is a North Countryman by birth and association, for he not only received his medical education at Edinburgh University, but subsequently held a lectureship in physiology at Durham. It is characteristic of the activity of certain senior surgeons on their retirement, that when he reached the retiring age in 1905 he was one of those who worked most energetically for the new buildings which were opened in the following year, and of which a bird's-eye view is given in the illustration on page 323. These facts are sufficient to indicate that Mr. Hume's paper, entitled "The Voluntary Hospital: on its Trial," will represent half a century's experience on the part of a hospital officer in the light of which our existing problems should be illuminated.

The deputy chairman of the Conference is Sir George Hare Philipson, M.D., D.C.L., J.P., the chairman of the infirmary house committee.

Sir George has had a very distinguished career, and a many-sided life, the enthusiasms of which are hardly lessened for all his seventy-eight years. A

COUNCILLOR HERBERT SHAW.

Newcastle man by birth, he received his education at University College, London, and at Caius College, Cambridge. Distinctions fell thick upon him. His degrees include those of M.A., M.D., LL.D., and he is a Fellow of the Royal College of Physicians and a member of the General Medical Council. He has been, moreover, President of the British Medical Association, Professor of Medicine

MR. WM. STRAKER.

at Durham University since 1876, and since 1912 he has been its Vice-Chancellor. In 1900 he received his knighthood, and he is known in the county as an amateur of the fine arts, of which he has always been a discriminating student.

Another notable personality at the Conference is Sir Riley Lord, who is chairman of the finance committee of the Royal Victoria Infirmary. A

Lancastrian by birth, and now seventy-six years old, he entered public life in Newcastle in 1885, became sheriff seven years later, and has held the office of Mayor on two occasions. During his first mayoralty, in 1895, he inaugurated the Victoria Diamond Jubilee Fund to build a new infirmary. The effects of this fund were even larger than its immediate result, which was £100,000, for Lord Armstrong, whom we have already alluded to in connection with Mr. Roden Orde, contributed a similar amount, and a third sum of six figures was received under the will of the late John Hall. This brings us back to the Royal Victoria Infirmary, of which, as the outcome of these efforts, the foundation-stone was laid by the Prince of Wales on June 20, 1900—exactly to a day fourteen years ago. The infirmary itself was opened by the King in July 1906.

## Figures and Tables

**Figure f1:**
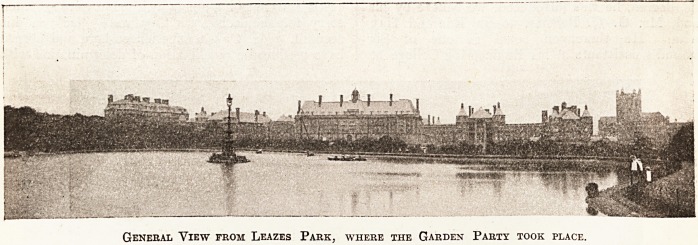


**Figure f2:**
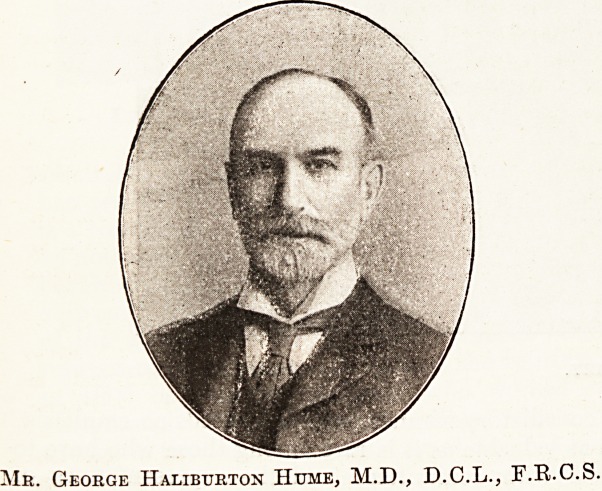


**Figure f3:**
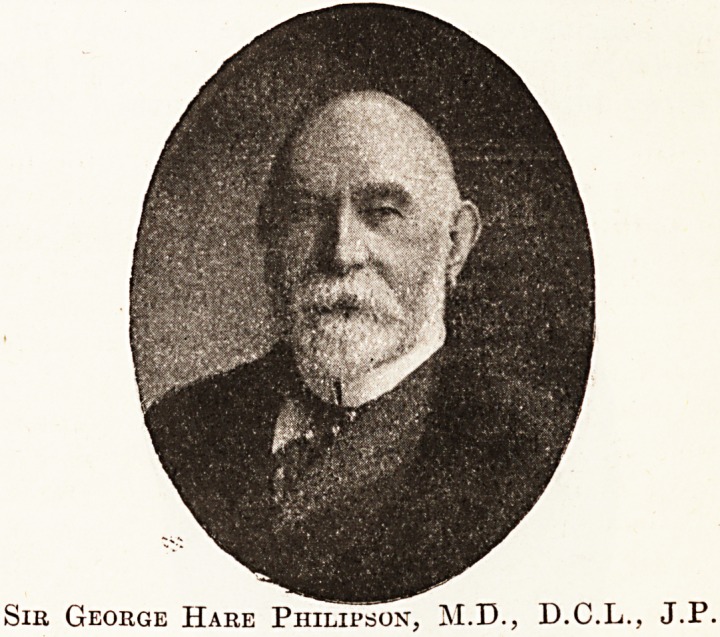


**Figure f4:**
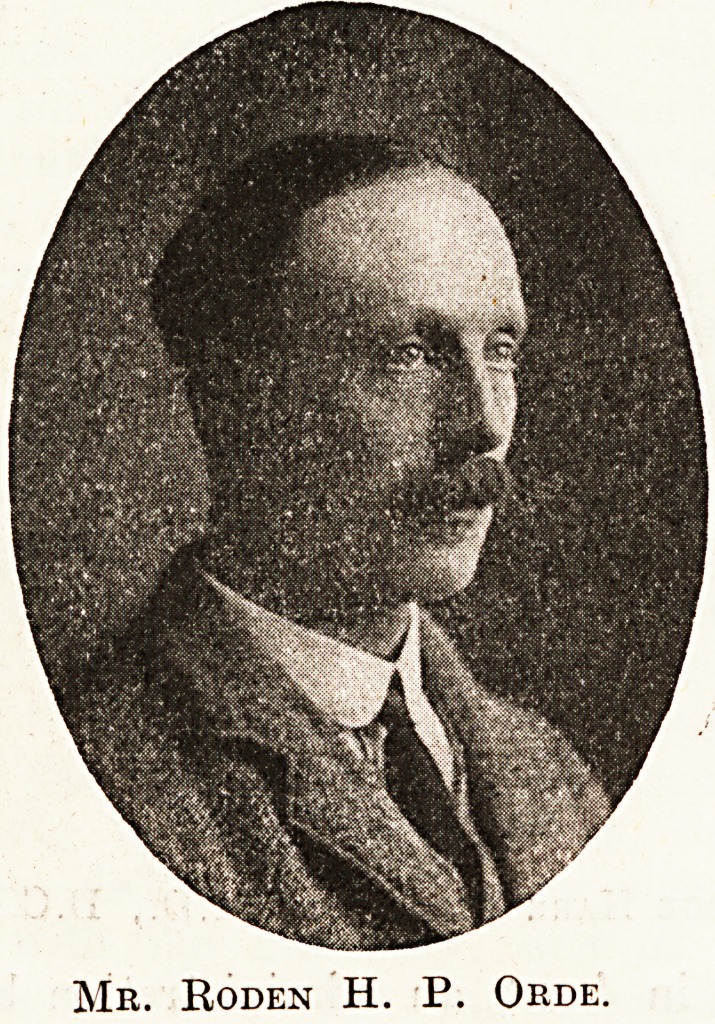


**Figure f5:**
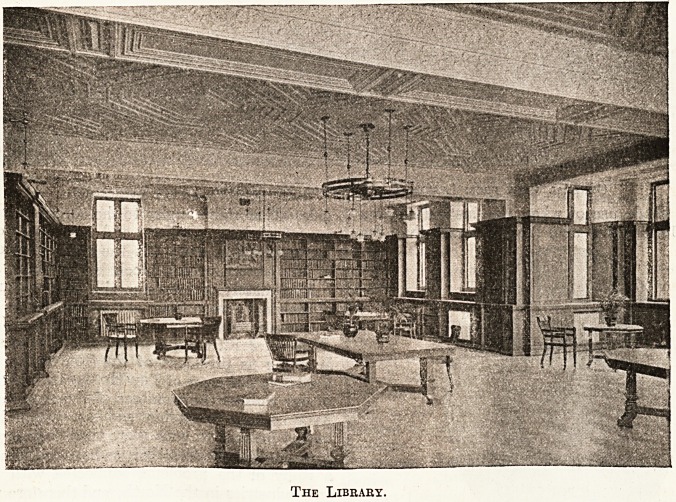


**Figure f6:**
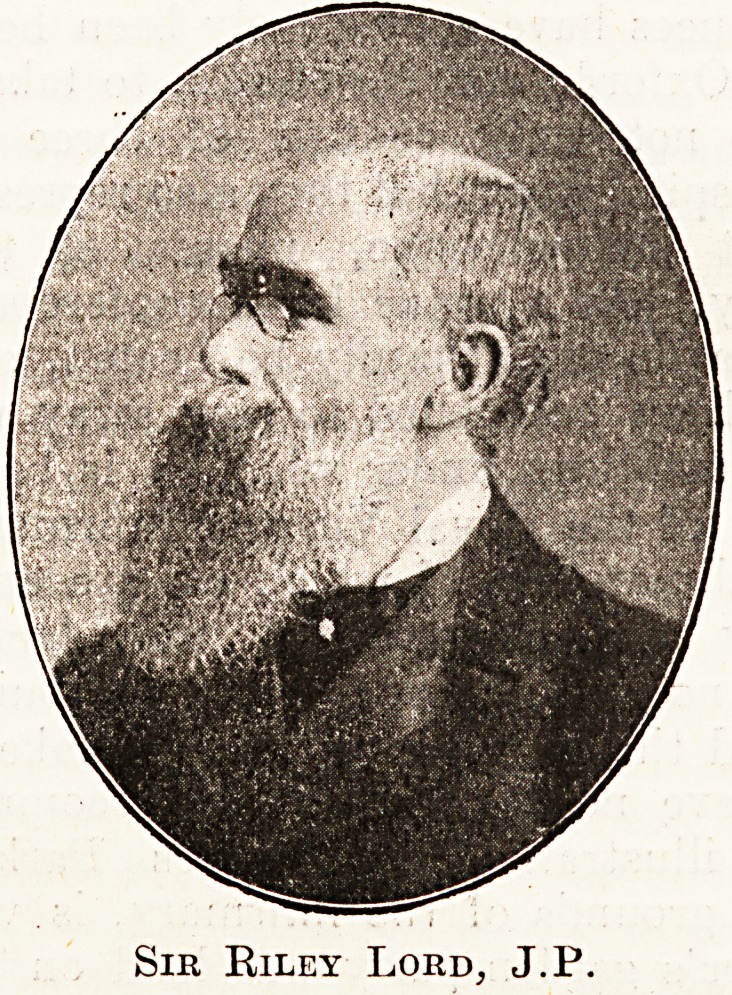


**Figure f7:**
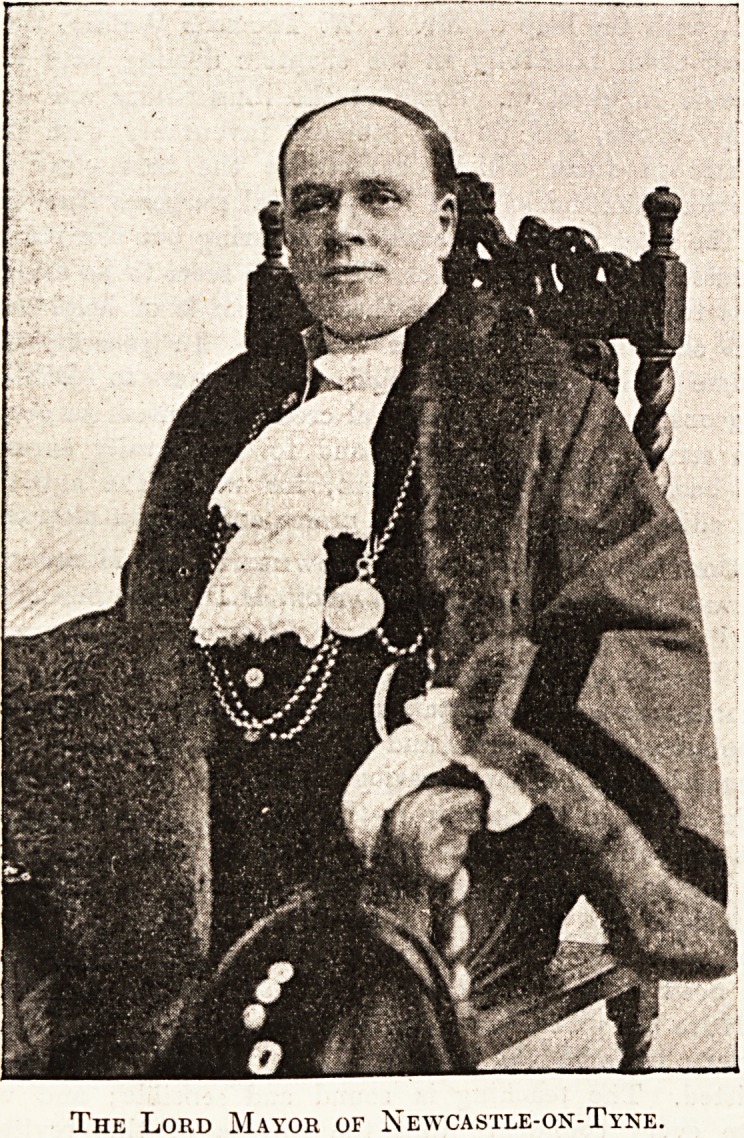


**Figure f8:**
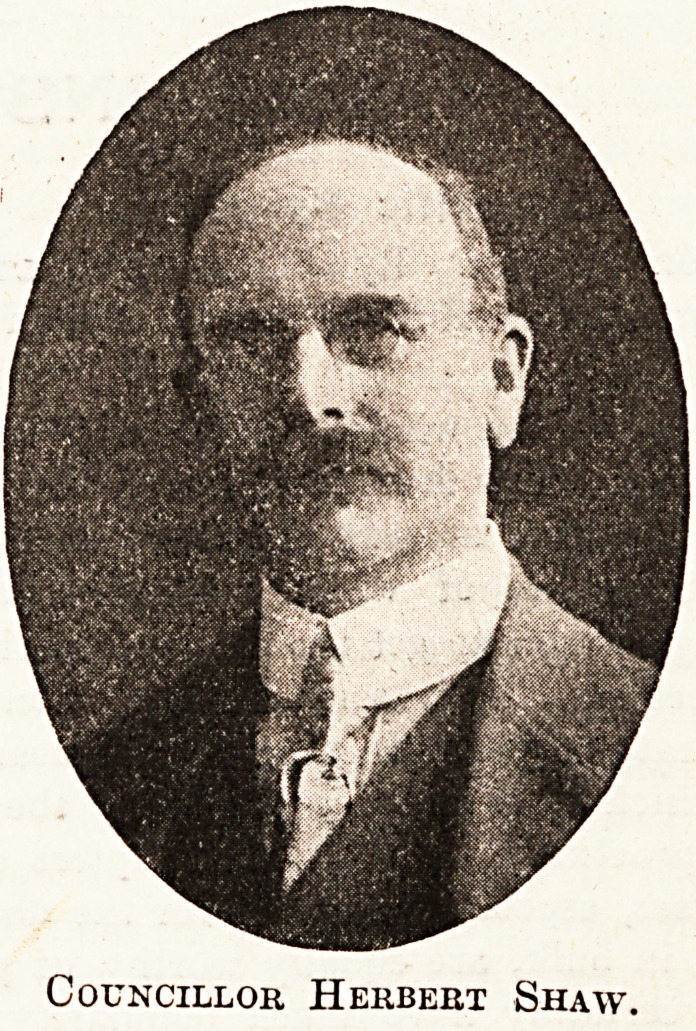


**Figure f9:**